# Book Review Computational Psychometrics: New Methodologies for a New Generation of Digital Learning and Assessment

**DOI:** 10.1007/s11336-022-09860-y

**Published:** 2022-04-08

**Authors:** Esther Ulitzsch

**Affiliations:** IPN—Leibniz Institute for Science and Mathematics Education, Olshausenstraße 62, 24118 Kiel, Germany

Innovations in technology-enhanced learning and assessment systems have evolved rapidly in recent years. Facilitating the (real-time) collection of process data that provide detailed documentation of how test takers and learners engage with the administered tasks—such as keystrokes, mouse movements, clickstreams, or audio traces of subjects interacting with each other—technology-enhanced learning and assessment systems entail vast opportunities. These range from a deepened understanding of how persons interact with the administered tasks through the assessment of capabilities that manifest themselves more in the process than in the final outcome—such as problem-solving strategies, collaboration, or inquiry—to the possibility to dynamically adapt the systems as well as ones’ beliefs on the targeted capabilities as test takers’ and learners’ interactions with the tasks evolve. How evidence on test takers’ and learners’ capabilities can be extracted and synthesized from this usually enormous and possibly unstructured amount of data, however, is not self-evident. In fact, many characteristics of process data violate the very assumptions of traditional psychometric models, such as unidimensionality or local independence.

To accommodate the sequential and unstructured nature of process data and leverage its potential, the *computational psychometrics* paradigm calls for enriching theory-grounded psychometrics with stochastic process theory and data-driven techniques originating in computer science (von Davier, 2017). The volume “Computational Psychometrics: New Methodologies for a New Generation of Digital Learning and Assessment” edited by Alina A. von Davier, Robert J. Mislevy, and Jiangang Hao (2021) provides an introduction and overview of the current state of the art of this newly emerging field of research and its methodologies. The volume targets an audience with a traditional psychometric background that is interested in technology-enhanced learning and assessment systems and aims to expand its toolbox with techniques that support handling the complex data such systems provide and/or is in search for guidelines and inspiration for planning such assessments and capitalizing on their opportunities. As the discussed methods are quite sophisticated and descriptions are oftentimes rather technical, readers with advanced psychometric and statistical training on the graduate level and good knowledge of R and/or Python will benefit most from this edited volume.

## Content of the Volume

The volume comprises 14 standalone chapters. Chapter 1, written by the volume’s editors, provides a general introduction into the field and the volume’s content. The volume is then organized into two parts. Part I (“Conceptualization”, Chapter 2–5) aims to provide the theoretic foundations for computational psychometrics and familiarizes readers with prominent types of technology-enhanced learning and assessment systems. Part II (“Methodology”, Chapter 6 –14) provides an overview of various techniques originating in fields outside of psychometrics that facilitate handling complex, unstructured data, examples of how these have been used in psychometric applications, and examples of R and Python code for their implementation. The code is embedded and commented in the chapters, and reproducible examples are made available in a Git repository accompanying the volume.^1^ Existing psychometric applications for each of the discussed techniques are referenced, which may be used as starting points for further reading. The methodology chapters aim at giving the reader a sound intuition for a wide range of methods that are not part of typical psychometric training and provide starting points for their implementation, rather than giving comprehensive tutorials.

Part I starts with Chapter 2, which discusses the potential of complex performance assessments for gathering evidence on capabilities that manifest themselves in interactions with unfolding situations. Taking an evidence-centered design perspective (Mislevy, Almond, & Lukas, 2003), it is further highlighted that valid evidence from process data can only be extracted when the tasks really elicit the targeted capabilities and support to record relevant data. Chapter 3 provides a more detailed overview of the computational psychometric framework, its relationship to the changing nature of educational measurement, and its rich and complex data, and delineates how bringing together psychometric and computational models may accommodate the specific challenges and tap the potentials that these data entail. Chapter 4 provides an introduction to virtual performance-based assessments. Central features of scenario-based, simulation-based, game-based, and collaborative assessments are discussed. The challenges of identifying and synthesizing evidence on the targeted capabilities from test takers’ activities on the tasks are discussed from design and analysis perspectives. Chapter 5 provides an introduction to adaptive learning systems. It reviews techniques such as Bayesian knowledge tracing or the Elo rating systems that aid the systems to update beliefs on students’ current states of knowledge and provides an intuition on how adaptive learning systems adapt to student attributes. Various examples for current adaptive learning systems are provided.

Part II starts off with Chapter 6, which reviews basic psychometric concepts, quality criteria, and models and discusses how this “psychometric backbone” can be carried over and extended to new types of assessments. Chapter 7 provides an introduction to Bayesian inference using Markov chain Monte Carlo (MCMC) techniques and illustrates how these can be used to estimate a broad range of computational models. Chapter 8 gives an introduction into techniques for handling complex data with Python, reviewing the parsing of unstructured process data, pipelines for large data files, and stream processing (i.e., analyzing a continuous data stream generated or loaded in real time). Chapter 9 provides an overview over canonical supervised machine learning techniques, such as support vector machines and gradient boosting. Among others, these can be used for classification problems. The utility of these techniques for psychometric applications is illustrated based on the automated scoring of chats from an online collaborative problem-solving study. Chapter 10 reviews common clustering algorithms such as *k*-means and DBSCAN, and dimensionality reduction approaches such as principal component analysis and multidimensional scaling. For illustration, the approaches are used for extracting parsimonious descriptions in terms of clusters or latent dimensions of solution strategies based on action sequence data from a problem-solving task. Chapter 11 gives an introduction into the basic concepts of more recent advances in machine learning such as deep neural networks and generative adversarial networks. Among others, the chapter references applications of deep neural networks for recognizing emotions in classroom video data or building course recommender systems for online learning platforms. Generative adversarial networks, which, intuitively speaking, can be employed to generate new data that resemble the data employed to train the networks, have yet not found much application in educational science and psychometrics. The chapter discusses their utility for modeling the actions, speech, or facial expressions of computer agents in collaborative assessment and learning environments. Chapter 12 gives examples of how the sequential nature of behavioral pathways through interactive systems may be modeled with time series and stochastic process models. Introductions to Markov decision process, state-space, and temporal point process models are provided. These models are well suited for modeling decision process, time series, and time-to-event data. For each model class, the chapter discusses its potential for psychometric applications, e.g., for modeling decisions in dynamic assessment and learning environments, the development of abilities, or time-stamped action sequence data. Chapter 13 discusses visualization tools and measures originating in social network analysis for describing typical behavioral pathways of test takers’ interactions with a task. Finally, Chapter 14 covers tools from natural language processing. It reviews feature construction, i.e., extraction of variables from text, and topic modeling as basic text mining concepts. It then discusses the use of natural language processing techniques for automated scoring of essays and constructed responses.

## Conclusion

The edited volume provides a great introduction into the necessity, constituting parts, theoretic foundations, and techniques of the newly emerging field of computational psychometrics. I see the major contribution of this volume to be threefold: First, it provides a comprehensive description of the opportunities as well as the challenges that technology-enhanced learning and assessment systems entail and contrasts these against classical static assessment systems and their relatively simple data structures that most traditional psychometric techniques have been developed for. Second, it familiarizes psychometricians with a broad array of concepts and techniques from stochastic process theory, computer science, and machine learning and showcases how integrating these with psychometrics can unleash the potential and meet the challenges of the vast amount of data gatherable from technology-enhanced learning and assessment systems. Third, and maybe most importantly, it delineates that successfully leveraging techniques originating in computer science and machine learning can only be achieved when grounded on—as opposed to replacing—the theory-based reasoning of psychometrics.

The method chapters provide a good overview and intuition of the discussed techniques and reference psychometric applications and standard literature that readers may turn to for further reading. To complement these standard references, future editions may consider compiling reading lists with introductory material tailored to psychologists and psychometricians (such as the excellent introduction into deep learning given by Urban & Gates, 2021). To even better grasp their utility for tackling various types of measurement problems, I would have found it helpful to have a greater emphasis on case studies that outline best practices of integrating the discussed techniques with psychometrics. While all method chapters mention various potential psychometric applications, there is some variety in the degree to which these are spelled out. The provided code varies in the extent to which it is commented. Some code snippets, such as the MCMC samplers discussed in Chapter 7 or the exemplary generative adversarial network implemented in Chapter 11, come with only few guidance, such that readers less familiar with the discussed techniques may find it difficult to directly use this code to get started and adapt this to investigate their own data.

The volume also illustrates that computational psychometrics is just emerging. While an impressive body of work has already accumulated, there are yet no canonical procedures for the field’s most pressing problem statements, e.g., a standard workflow to follow for extracting, synthesizing, and validating evidence from process data. The method chapters and the references provided therein serve as excellent starting points and inspiration for researchers in search of innovative solutions for their measurement problems and further advancing this rapidly evolving field, but do not yet provide definite answers.

## Footnotes

1 https://github.com/jgbrainstorm/computational_psychometrics.
